# Aerobic exercise training attenuates cardiac inflammation and fibrosis in mice with type 2 diabetes and inhibits the advanced glycation end products pathway

**DOI:** 10.1186/s13098-025-02076-x

**Published:** 2026-01-07

**Authors:** Karine Lino Rodrigues, Vivian Vieira Dias Da Silva, Daniel Olindo de Castro-Linhares, Evelyn Nunes Goulart da Silva Pereira, Raquel Rangel Silvares, Beatriz Peres de Araujo, Juliana Magalhães Chaves Barbosa, Anissa Daliry

**Affiliations:** 1 Laboratory of Clinical and Experimental Physiopathology, Oswaldo Cruz Foundation, Rio de Janeiro, Brazil; 2 Cellular Biology Laboratory, Oswaldo Cruz Institute, Rio de Janeiro, Brazil

**Keywords:** Aerobic training, Advanced glycation end products, Type 2 diabetes, Cardioprotection

## Abstract

**Background:**

Type 2 diabetes mellitus (T2D) is associated with cardiac dysfunction caused by oxidative stress, inflammation, and fibrosis. Exercise has shown cardioprotective effects in T2D. However, the impact on the Advanced Glycation End Products (AGE) and its receptors remains unclear. In this study, we investigated whether aerobic exercise modulates the AGE signaling pathway in the hearts of diabetic mice and whether it is associated with oxidative and inflammatory damage.

**Methods:**

Male C57BL/6 mice were fed a control (CTL) diet or a high-fat, high-carbohydrate (HFHC) diet to induce T2D. A subset of the T2D mice underwent aerobic training for 12 weeks (T2D EX), whereas the other mice remained sedentary (T2D). Cardiac tissues were analyzed for AGE deposition, AGE receptors expression, oxidative stress markers, cytokine profiles, and histological changes, including fibrosis and inflammation.

**Results:**

Aerobic exercise in T2D mice reduced the cardiac deposition of fluorescent AGEs and CML, decreased RAGE protein and gene expression, downregulated CD36 and galectin-3 receptors, while not affecting GLO-1 detoxification system. Exercise in T2D mice suppressed cardiac inflammation and fibrosis. Improvements in inflammatory profiles included reduced expression of IL-6, TNF-α, and NF-kB. However, markers of oxidative stress, such as malondialdehyde, remained largely unaffected by exercise. Pearson’s correlation analysis showed strong associations between AGE signaling pathway components and cardiac fibrosis, inflammation, and oxidative stress parameters.

**Conclusions:**

Aerobic exercise mitigates cardiac changes in T2D by downregulating the AGE signaling pathway and reducing fibrosis and inflammation. These findings highlight the therapeutic potential of exercise in interfering with AGE-mediated mechanisms to alleviate T2D-associated cardiovascular complications.

**Supplementary Information:**

The online version contains supplementary material available at 10.1186/s13098-025-02076-x.

## Background

Type 2 diabetes (T2D) is a chronic multifactorial disease that affects over 400 million peopleworldwide [1]. T2D is characterized by pancreatic insufficiency and insulin resistance. The resulting hyperglycemia is the main cause of numerous life-threatening complications, including cardiovascular disease, neuropathy, nephropathy, retinopathy, and an increased risk of infection [2, 3]. People with T2D are more prone to cardiovascular complications, which are the most common cause of death in these patients [4, 5]. However, the exact mechanism that triggers the development of cardiovascular complications in T2D is not yet fully understood. Efforts to characterize the underlying mechanisms are essential to prevent or at least delay the fundamental biological processes that cause diabetic heart disease.

Advanced glycation end products (AGEs) and advanced lipid peroxidation end products (ALEs) have been identified as possible pathways involved in the etiology and development of diabetes [6, 7]. AGEs result from the non-enzymatic glycation of proteins, lipids, or nucleic acids by reducing sugars such as glucose. ALEs, result from the oxidative degradation of polyunsaturated fatty acids, producing reactive aldehydes that modify biomolecules. AGEs/ALEs accumulate in tissues due to chronic metabolic changes such as hyperglycemia and oxidative stress, which are exacerbated in diabetes [8, 9]. These compounds exert their harmful effects mainly by binding to specific receptors expressed on various cell types, including endothelial cells and cardiomyocytes [10]. The receptor for Advanced Glycation End Products (RAGE) is a multiligand cell surface receptor that plays a central role in transmitting AGE-induced signaling, leading to the activation of pro-inflammatory transcription factors such as nuclear factor kappa B (NF-κB) [11, 12]. CD36 is another receptor involved in lipid uptake and oxidative stress. CD36 also interacts with AGEs/ALEs and contributes to lipotoxicity in cardiomyocytes, further promoting inflammation and metabolic dysfunction [13, 14]. Galectin-3, a β-galactoside-binding lectin, also binds AGEs and contributes to inflammation and fibrosis through alternative signaling pathways [15, 16] Glyoxalase 1 (GLO1) is a detoxifying enzyme that degrades methylglyoxal, an important precursor of AGEs [17]. Reduced GLO1 activity is associated with increased accumulation of AGEs and glycation stress, while its upregulation is considered a protective mechanism in hyperglycemia [18].

In the endothelium, activation of the AGE-RAGE axis leads to decreased nitric oxide (NO) bioavailability, increased production of reactive oxygen species (ROS) and impaired vasodilation, contributing to endothelial dysfunction and vascular stiffness [19–26]. Additionally, studies show that the interaction of AGEs/ALEs with extracellular matrix proteins contributes to vascular stiffening and myocardial dysfunction, while myofilament glycation decreases cardiac contractility by inhibiting tropomyosin movement [27–29]. These structural changes are associated with increased expression of profibrotic cytokines such as transforming growth factor-beta (TGF-β), contributing to interstitial fibrosis and maladaptive cardiac remodeling [30]. In cardiomyocytes, AGEs also directly glycate key contractile proteins, including actin and myosin, leading to decreased calcium sensitivity and impaired excitation-contraction coupling [31, 32]. These molecular and cellular changes result in reduced systolic function and contribute to the progression of diabetic cardiomyopathy. AGEs/ALEs can also induce autophagy and apoptosis in cardiomyocytes, in addition to causing structural and functional changes that exacerbate cardiovascular dysfunction and impair cardiac contractility [33–35]. This is mediated by increased oxidative stress and mitochondrial dysfunction, as well as activation of pro-apoptotic signaling pathways [35]. AGEs binding to RAGE on cardiomyocytes also leads to chronic activation of NF-κB and mitogen-activated protein kinase (MAPK) signaling pathways, maintaining a pro-inflammatory and pro-fibrotic environment. These cumulative effects contribute to cardiomyocyte loss, impaired cardiac performance and increased susceptibility to heart failure in diabetes.

Exercise is widely recognized as essential for combating metabolic diseases such as T2D [36, 37]. Exercise can be divided into several categories, with the most important being aerobic (long-duration exercise that increases cardiovascular endurance) and anaerobic (high-intensity exercise for short periods that develops strength and muscle mass) [38, 39]. Regular aerobic exercise of moderate to vigorous intensity plays a fundamental role in combating T2D, by promoting blood glucose regulation and improving β-cell function. It also improves insulin sensitivity, lowers fasting blood glucose and glycated hemoglobin (HbA1c), and reduces body weight and visceral adiposity [37, 40, 41]. Studies show that aerobic exercise training helps lower blood pressure and improve the lipid profile by increasing HDL levels and lowering LDL and triglyceride levels - factors crucial for cardiovascular health [42–45]. Aerobic exercise training increases blood flow and improves endothelial function. It also enhances the body’s antioxidant capacity, helping to neutralize of free radicals [46–48]. These metabolic benefits are mediated, at least in part, by increased glucose uptake in skeletal muscle, upregulation of proteins related to lipid metabolism, and decreased insulin resistance in peripheral tissues. In addition, aerobic training exercise has been reported to reduce the accumulation of AGEs and decrease RAGE expression in target tissues, which may contribute to improved vascular and metabolic function [46, 49]. Our group previously, reported a reduction in liver-specific RAGE expression in diet-induced T2D mice after aerobic exercise, while Malin et al. (2020) reported changes, particularly in circulating soluble RAGE, in adults undergoing a combined lifestyle intervention, including aerobic exercise and caloric restriction, which were associated with an improved glucose tolerance and insulin sensitivity [46, 50]. Wang et al. (2023) also showed that long-term voluntary exercise training inhibited AGE/RAGE signaling and decreased microglial activation in a mouse model of Alzheimer’s disease, suggesting possible neuroprotective effects [51]. Overall, these results suggest that exercise may play a role in counteracting AGE/ALE accumulation and RAGE signaling. However, the precise effects of exercise on cardiac AGE signaling – including ligands, receptors, and the detoxification system – as well as the downstream effects of pathway activation, such as inflammation and oxidative stress, remain poorly characterized.

Given evidence that the AGE–RAGE signaling pathway plays a significant role in the pathophysiology of diabetes and that exercise is an effective non-pharmacologic strategy to reduce its metabolic and cardiovascular complications, the aim of this study was to determine whether modulation of the AGE–RAGE signaling pathway in the cardiac tissue of diabetic mice contributes, at least in part, to the molecular mechanisms by which exercise training can attenuate diabetes-associated cardiac dysfunction. Additionally, we examined whether modulation of the AGE–RAGE signaling pathway is related to changes in oxidative and inflammatory damage in the cardiac tissue of diabetic C57BL/6 mice. Our study underscores the importance of the AGE–RAGE signaling pathway in diabetes-related cardiac changes and suggests that this pathway is a promising therapeutic target.

## Methods

### Experimental protocol and animals

**Fig. 1:**
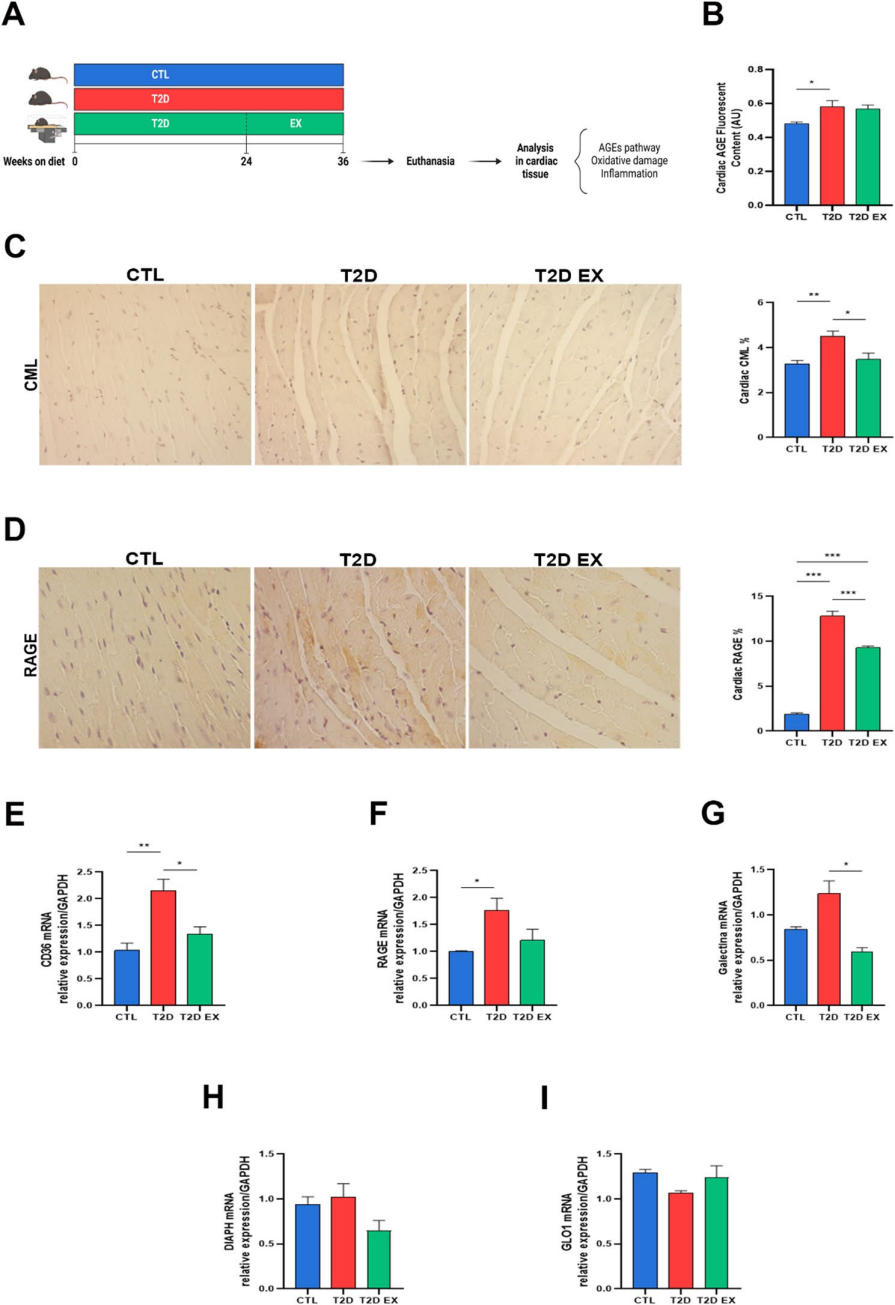
Effect of exercise on the pathway of advanced glycation end products pathway in cardiac tissue of T2D mice. Study design (**A**), mice were fed a control diet (CTL) or a high-fat, high-carbohydrate diet (HFHC) to induce type 2 diabetes (T2D). **A**, subset of HFHC-fed mice was subjected to a 12-week aerobic exercise treining protocol (T2D EX); Quantification of fluorescent AGE levels in cardiac tissue (**B**) (*n* = 10 biological replicates, using a fluorescence-based assay); Representative images and quantification of protein expression of CML and RAGE (**C** and **D**) in cardiac tissue (*n* = 5 biological replicates, using immunohistochemistry); real-time PCR analyzes showing mRNA transcript levels of CD36, RAGE, Galectin-3, DIAPH1 and GLO1 genes in cardiac tissue (**E**–**I**) (*n* = 6 biological replicates, using quantitative PCR). *P < 0.05; **P < 0.01; ***P < 0.001

C57BL/6 mice (8 weeks old) from the central vivarium of the Oswaldo Cruz Foundation, Brazil, were used. Mice were housed in standard cages with controlled temperature (± 22 °C) and 12-hour light-dark cycles (darkness from 6 pm). T2D was induced by a high-carbohydrate, high-fat diet, and mice were assigned to three groups: CTL (control diet, sedentary, *n* = 10), T2D (high-carbohydrate, high-fat diet with 25% fructose, sedentary, *n* = 10), and T2D EX (high-carbohydrate, high-fat diet with 25% fructose, exercised, *n* = 10). The exercise protocol, previously standardized by Rodrigues et al. (2022), consisted of treadmill running three times per week for 30 min each session over 12 weeks [46]. At the end of the protocol (36 weeks), all mice underwent deep anesthesia, followed by cardiac puncture for whole blood sampling and immediate euthanasia. Blood was carefully collected from the right ventricle in a single pass using a 25 G needle to avoid tissue damage; this tissue was used for subsequent histologic and molecular analyses. The heart was then carefully excised, and the left ventricle was processed for all subsequent analyses. Serum was collected by centrifugation at 700×g for 15 min at 4 °C, and aliquots were stored at −80 °C for future analyses. The overall experimental design and schedule are shown in Fig. 1. The mice used in this study are the same cohort previously reported in Rodrigues et al. (2022) [46], where T2D was thoroughly validated using fasting glucose levels, dyslipidemia, oxidative stress parameters, inflammatory cytokine profiles, and vascular function. The current work provides a complementary analysis focusing on molecular and histologic markers of cardiac damage and cardioprotection in a mouse model of T2D. Mice were randomly assigned to experimental groups (CTL, T2D, T2D EX), and the researchers responsible for data collection and analysis were blinded to the experimental group. All experiments were conducted in accordance with the internationally recognized standards described in the Guide for the Care and Use of Laboratory Animals (National Research Council, USA). These procedures were approved by the Animal Welfare Committee of the Oswaldo Cruz Foundation under license L-0012/18 A1 to ensure the highest ethical and welfare standards.

Although it is known that in diet-induced obesity (DIO) models some animals may remain resistant to obesity on a high-fat diet, in our protocol, all animals in the T2D group developed obesity; therefore, no exclusions were necessary. The final number of *n* = 10 per group reflects both the baseline and terminal cohorts.

Each of the following subsections describes the type of samples used (serum or tissue) and the corresponding methods employed to achieve the study objectives.

### Aerobic exercise training

Aerobic exercise training started at week 24 and continued for 12 weeks. The mice exercised on a treadmill (Hectron Fitness Equipment, Brazil) with an incline of 0, at 80% of their maximal speed (approximately 75–80% of maximal oxygen uptake) for 30 min, three times per week [46].

### Cardioprotective index (CPI)

The cardioprotective index (CPI) of the mice was calculated as the HDL-c concentration (mmol/L) divided by the LDL-c concentration (mmol/L) [52]. Measurements were performed using serum samples from seven animals per group and determined with commercially available kits (Bioclin System II, Belo Horizonte, Brazil) according to the manufacturer’s instructions.

### Histopathology

Fragments of the left ventricle were collected and fixed in 4% w/v formaldehyde in 0.1 M phosphate buffer, pH 7.2. After processing, they were embedded in paraffin and cut into 5 μm thick sections. The sections were stained with hematoxylin and eosin (HE) and examined under a light microscope (Nikon model 80i; DSRi1 digital camera, Nikon Instruments, Inc., Melville, NY, USA). Inflammatory infiltration was qualitatively assessed by light microscopy, focusing on the presence, distribution, and density of inflammatory cell aggregates in the interstitial and perivascular regions of the myocardium. Criteria included changes in tissue architecture, localization of inflammatory foci, and morphological identification of infiltrating cells (predominantly mononuclear), based on nuclear size, shape, and cytoplasmic features. Masson’s trichrome staining was used to detect and evaluate fibrosis by quantifying collagen with ImageJ software (ImageJ, Burleson, TX, USA). Four histologic sections from five animals per group were examined.

### Measurement of advanced glycation/lipoxidation end products

The concentrations of fluorescent AGEs in the heart were measured by spectrofluorimetric detection according to the method described by Nakayama [53]. Heart tissue fractions were incubated overnight in a chloroform: methanol solution (2:1, v/v) for lipid extraction. The samples were washed three times with methanol and three times with 0.1 N NaOH to remove residual solvent. The tissue was then homogenized in 0.1 N NaOH, followed by centrifugation at 8,000 × g for 14 min at 4 °C, and the supernatant was collected. The fluorescence of the AGE samples was recorded at an emission wavelength of 440 nm and an excitation wavelength of 370 nm, using 0.1 N NaOH as a blank. Measurements were performed using a SpectraMax Plus Reader (Molecular Devices, San Jose, CA, USA). A native bovine serum albumin (BSA) solution (1 mg/mL in 0.1 N NaOH) was used as a reference, and its fluorescence intensity was defined as the fluorescence unit. The fluorescence values of the samples were determined at a protein concentration of 1 mg/mL relative to the native BSA reference. The tests were performed with ten biological replicates per group.

### Immunohistochemistry

After deparaffinization, heart-sections were treated with 3% H_2_O_2_ and blocked with a solution of 5% skimmed milk powder and 5% BSA. Sections were then washed with PBS and incubated overnight in a humidified chamber at 4 °C with primary monoclonal antibodies against mouse RAGE (1:200, sc-365154; Santa Cruz Biotechnology, Santa Cruz, CA, USA) and carboxymethyllysine (CML) (1:200, ab125145; Abcam, Cambridge, UK). After washing with PBS, the sections were treated with biotinylated secondary antibodies, followed by incubation with peroxidase-conjugated streptavidin. Diaminobenzidine (DAB) was used as the chromogenic substrate. Finally, the sections were counterstained with Mayer’s hematoxylin. Immunostaining was quantified using ImageJ software. The analysis was performed on four sections from five animals per group.

### Analysis of cytokines by flow cytometry

To assess the profile of cardiac inflammatory cytokines, we used the Cytometric Bead Array (CBA) Mouse Th1/Th2/Th17 Cytokine Kit (Becton Dickinson, Franklin Lakes, NJ, USA), which allows simultaneous quantification of multiple cytokines associated with Th1, Th2, and Th17 responses. This method is based on flow cytometry and uses fluorescent beads coated with specific antibodies for the following cytokines: interleukin-2 (IL-2), interleukin-4 (IL-4), interleukin-6 (IL-6), interferon- γ (IFN-γ), tumor necrosis factor-α (TNF-α), interleukin-17 A (IL-17 A), and interleukin-10 (IL-10). Cardiac tissue samples were thawed and lysed by sonication in a phosphate buffer containing a protease inhibitor cocktail (Roche, Basel, Switzerland) and Nonidet P40 (Sigma-Aldrich, Burlington, MA, USA) at a final concentration of 1%. The lysates were centrifuged at 500 × g for 5 min and the supernatant was removed. Subsequently, 25 µL of the samples, 25 µL of the bead capture mixture and 25 µL of the fluorochrome phycoerythrin (PE) were incubated for 2 h at room temperature. After incubation, the samples were washed with 500 µL wash buffer, centrifuged at 200 g for 5 min, and the supernatant discarded. The pellets were resuspended in 100 µL wash buffer. Data were collected using a Cytoflex flow cytometer (Beckman Coulter, Brea, CA, USA). Protein quantification of the cardiac tissue used for normalization was performed using the BCA Protein Assay Kit (Thermo Fisher Scientific, Waltham, MA, USA) according to the manufacturer’s instructions. Cytokine measurements were performed with six animals per group.

### Real-time reverse transcription polymerase chain reaction (RT-PCR)

Total RNA was extracted from heart tissue using the RNeasy Mini Kit (Qiagen, Hilden, Germany). cDNA synthesis was performed with the high-capacity cDNA reverse transcription Kit (Applied Biosystems, Waltham, MA, USA) using 1 µg of RNA, in a final reaction volume of 20 µL. Specific primers were used to amplify the following target genes: TNF-alpha, IL-6, NF-kB, RAGE, CD36, DIAPH1, GALECTIN-3, GLO1, and GAPDH (Table 1). RT-PCR was performed using power SYBR Green PCR Master Mix (Applied Biosystems) according to the manufacturer’s guidelines, on the 7500 fast platform (Applied Biosystems). Gene expression was quantified using the ∆∆Ct method and normalized to GAPDH expression. Analyses were performed with six biological samples per group.

**Table 1:** Primers used for PCR amplification in the study

Target		Sequence
TNF-α	Forward	5’-CTACCTTGTTGCCTCCTCTTT-3 ‘
	Reverse	5’-GAGCAGAGGTTCAGTGATGTAG-3 ‘
IL-6	Forward	5’-ACAACCACGGCCTTCCCTACTT-3 ‘
	Reverse	5’-CACGATTTCCCAGAGAACATGTG-3 ‘
NF-Kb	Forward	5’-GAAGTGAGAGAGTGAGCGAGAGAG-3’
	Reverse	5’-CGGGTGGCGAAACCTCCTC-3’
RAGE	Forward	5´ CAG GGT CAC AGA AAC CGG 3´
	Reverse	3´ ATT CAG CTC TGC ACG TTC CT 5´
CD36	Forward	5’CCTGGGAGTTGGCGAGAAA3’
	Reverse	5’CGATCACAGCCCATTCTCCT3’
DIAPH-1	Forward	5’-TGTCACCTCTGCTTTTCCTC-3’
	Reverse	5’-GAGAGTGGTTGAGACCCTTTG-3’
Gal-3	Forward	For 5’-CCCGCATGCTGATCACAATC-3’
	Reverse	Rev 5’-GGGGTTAAAGTGGAAGGCAA-3’
Glo-1	Forward	For 5’-CCCTGCTATGAAGTTCTCGCTC-3’
	Reverse	Rev 5’-GAGCTCAAGGGTGGCTTTTCT-3′
GAPDH	Forward	5′-CATGGCCTTCCGTGTTCCTA-3 ′
	Reverse	5′- GCGGCACGTCAGATCCA-3 ′

### Thiobarbituric acid reactive substance

Lipid peroxidation (LPO) in cardiac tissue was assessed by measuring thiobarbituric acid reactive substances (TBARS) using a spectrophotometric method based on malondialdehyde (MDA) concentration. The tissue was homogenized in cold phosphate-buffered saline (PBS) (pH 7.4) with butylated hydroxytoluene (BHT) at a final concentration of 0.2%. An equal volume of 0.67% thiobarbituric acid (Sigma Chemical Co., St. Louis, MO, USA) was added to a 0.5 mL sample, and each tube was sealed and incubated at 96 °C for 30 min. For spectrophotometric analysis, 200 µL of each sample was measured at 535 nm using a SpectraMax Plus Reader (Molecular Devices). Results were expressed as MDA, with an extinction coefficient of ε = 1.56 × 105 M − 1 cm – 1. TBARS measurements were performed with ten animals per group.

### Catalase (CAT) antioxidant activity

In cardiac tissue, the enzymatic decomposition of H_2_O_2_ was evaluated by homogenizing 100 mg of heart tissue in potassium phosphate buffer (KPE) and centrifuging at 600 ×g for 10 min at 4 °C. The supernatant was collected, and total protein concentration was quantified using the bicinchoninic acid (BCA) assay for normalization. The reaction mixture was prepared at a 25:4 (v/v) ratio and consisted of 50mM PBS (pH 7.4) and 0.3% H_2_O_2_. For the assay, 3.0 µL of the sample was added to 97 µL of the reaction mixture in each well of a UV-transparent 96-well plate, resulting in a final volume of 100 µL. Absorbance was measured at 240 nm over a 60-second interval (Δt = 30 s) using a SpectraMax Plus Reader (Molecular Devices). Enzymatic activity was expressed as units per milligram of protein (U/mg protein). CAT activity was measured using ten biological samples per group.

### Statistical analysis

The results are presented as mean values ± SEM for each group. The normality of variable distributions was assessed using the Shapiro–Wilk test and confirmed with Q-Q plots. Group comparisons were performed using one-way ANOVA, followed by the Bonferroni post hoc test. Pearson’s correlation analyses were conducted to evaluate the role of cardiac AGEs in the development of pathological cardiac changes in T2D. Specifically, correlations were examined between cardiac CML, RAGE expression, histological parameters (fibrosis and inflammatory infiltrate), oxidative stress markers (malondialdehyde, catalase activity, fluorescent AGEs), and cytokine levels. Data analysis was performed using GraphPad Prism 8.0.1 (GraphPad Software Inc., La Jolla, CA, USA). Statistical significance was defined as *p* < 0.05.

### Randomization and blinding

The mice were randomly assigned to experimental groups without stratification or blocking. For the exercise intervention, assignment to sedentary or aerobic exercise training was also randomized. To avoid environmental bias, cage positions, order of euthanasia and order of tissue removal were randomized. Primary outcomes were assessed under blinded conditions: slides and image files were coded, histology was assessed by two independent, blinded observers, and molecular tests were performed in randomized order on de-identified samples. Statistical analyses were performed on anonymized datasets, with group codes disclosed only after completion of the primary analysis. Personnel performing treadmill training were unblinded but had no influence on outcome measures or data analysis.

## Results

### Effect of aerobic exercise training on the pathway of advanced glycation end products in cardiac tissue in T2D

The T2D group showed increased cardiac deposition of fluorescent AGEs (Fig. 1B), along with higher protein expression of CML and RAGE (Fig. 1C-D) and elevated gene expression of CD36,RAGE and galectin-3 compared with the CTL group (Fig. 1E-F). Aerobic exercise training decreased the protein expression of CML and RAGE and reduced the gene expression of CD36 and galectin-3 compared to the T2D group (Fig. 1C-G). There were no significant differences in the expression of *DIAPH1* or *GLO1* among groups (Fig. 1H-I).

### Effect of aerobic exercise training on T2D-induced oxidative damage and inflammation of the heart

**Fig. 2:**
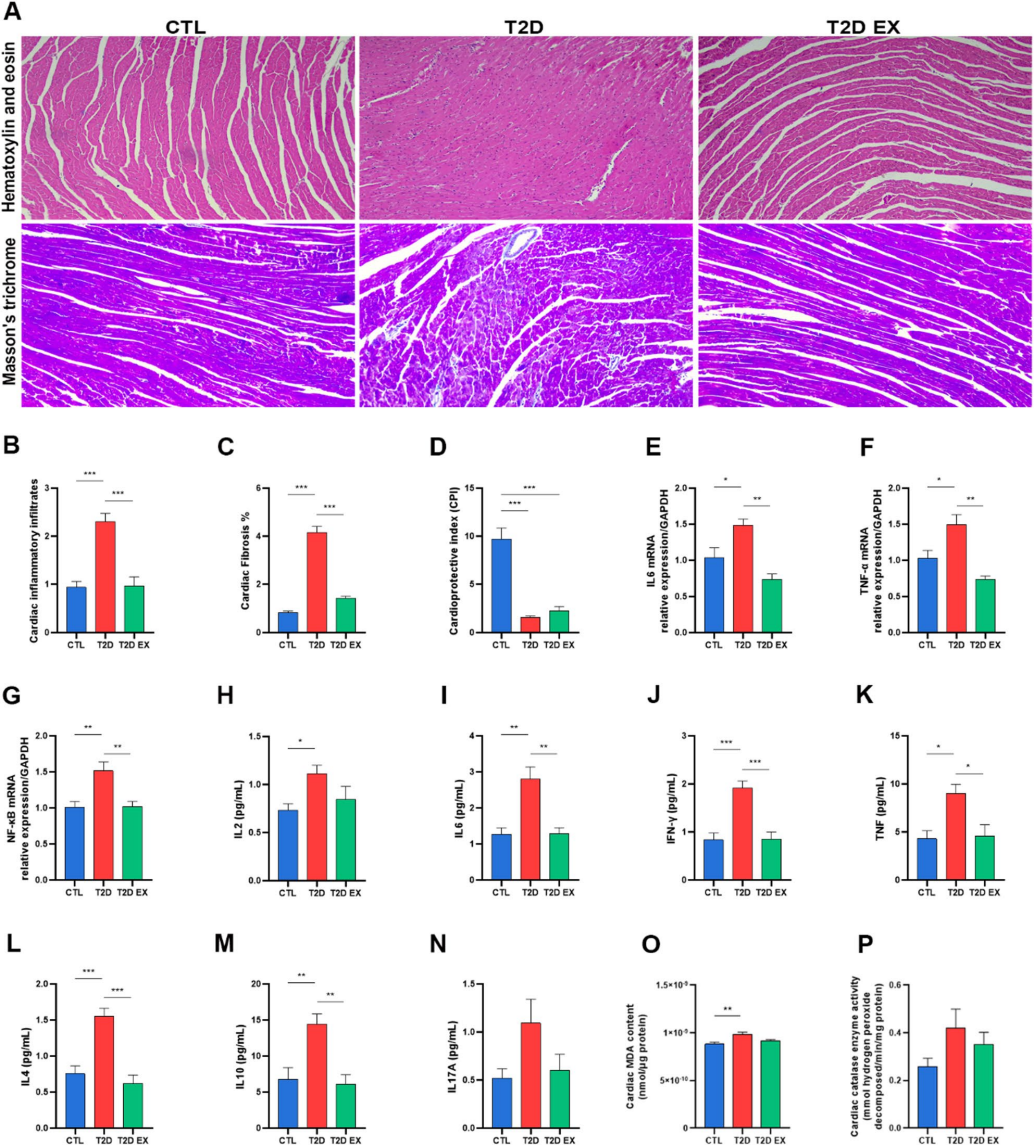
Effects of exercise on oxidative stress and cardiac inflammatory response in T2D. Representative images of cardiac samples stained with hematoxylin-eosin (H&E) and Masson’s trichrome (**A**); Quantitative assessment of inflammatory infiltrate (**B**) and fibrosis (**C**) (*n* = 5 biological replicates, using histopathology); Assessment of cardioprotective index (**D**) (*n* = 7 biological replicates, using HDL-c concentration divided by LDL-c concentration); Real-time PCR analyzes showing mRNA transcription levels of IL-6, TNF-alpha and NF-KB genes in cardiac tissue (**E**–**G**) (*n* = 6 biological replicates, using quantitative PCR); Quantification of Th1 (**H**-**K**), Th2 (**L** and **M**) and Th17 (**N**) cytokine and chemokine levels (*n* = 6 biological replicates, using quantitative flow cytometry); quantification of reactive thiobarbituric acid species, malondialdehyde (MDA) and catalase (CAT) enzymatic activity in the heart (**O** and **P**) (*n* = 10 biological replicates, using spectrophotometric analysis) of mice, fed a control diet (CTL) or a high-fat, high-carbohydrate (HFHC) diet to induce type 2 diabetes (T2D), with a subset of HFHC-fed mice subjected to a 12-week exercise protocol (T2D EX). *P < 0.05; **P < 0.01; ***P < 0.001

T2D mice showed a significant increase in inflammatory infiltration and fibrosis in cardiac tissue compared to the CTL group, as shown in Fig. 2A-C. In addition, a decrease in the cardioprotective index was observed in T2D mice compared to the CTL group (Fig. 2D). Aerobic exercise training effectively reduced both inflammatory infiltration and fibrosis in T2D mice. The T2D group showed increased gene expression of IL-6, TNF-alpha and NF-KB, as well as higher cytokine levels of IL-2, IL-6, IFN- γ, TNF, IL-4 and IL-10 compared with the CTL group, while aerobic exercise training decreased their levels, except for IL-2, which remained unchanged (Fig. 2E-N). IL-17 A cytokine levels did not differ between the groups (Fig. 2N).

Regarding oxidative damage in the cardiac tissue, T2D mice showed increased lipid peroxidation (LPO), with higher malondialdehyde levels compared to the control group (Fig. 2O). T2D also showed a trend toward increased activity of the antioxidant enzyme catalase; however, this increase was not statistically significant, and aerobic exercise training had no significant effect on these parameters (Fig. 2P).

### Correlation of AGE-RAGE pathway with cardiac remodeling, oxidative stress and inflammation in T2D

**Fig. 3:**
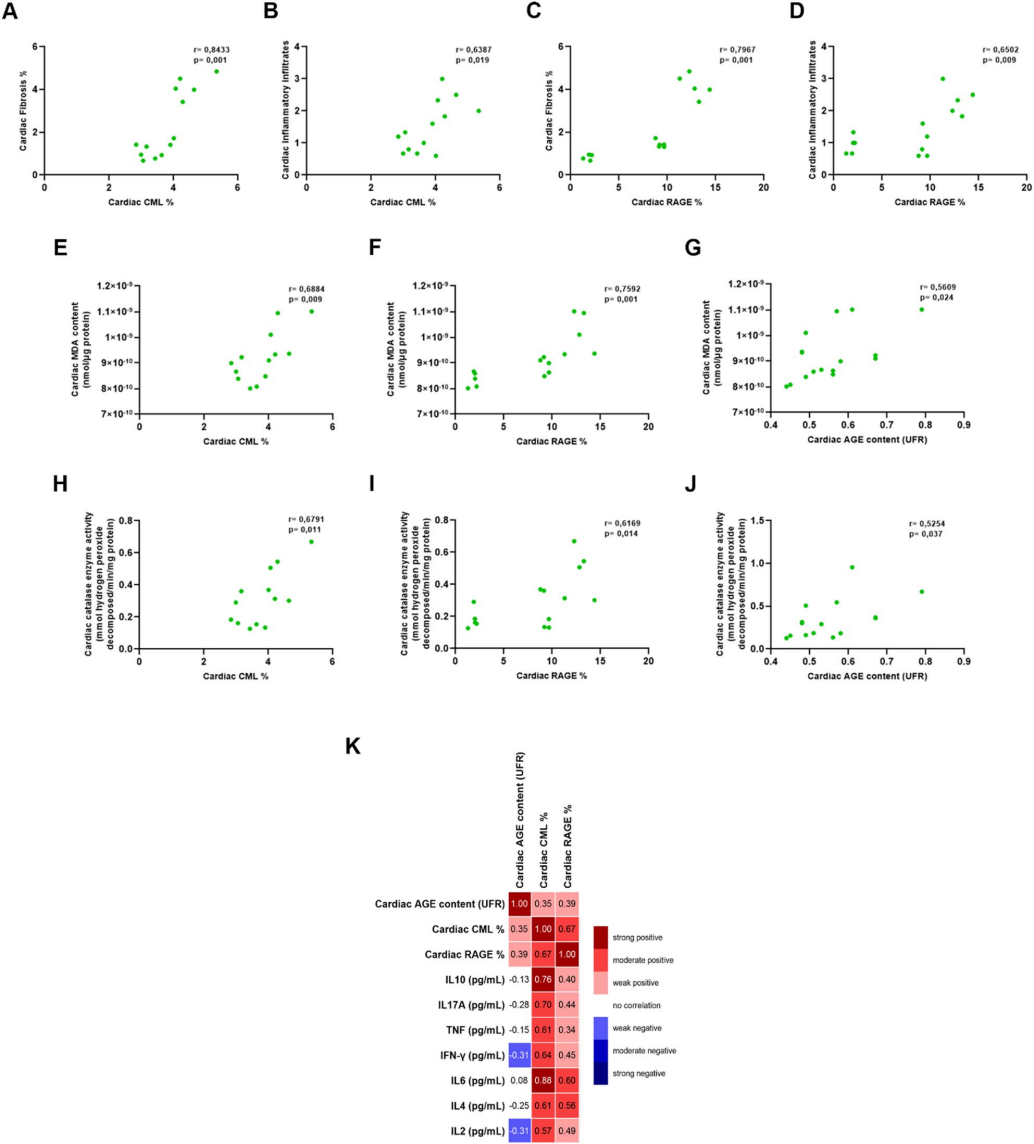
Correlation between cardiac AGEs, inflammation, and oxidative stress in T2D. Pearson correlation analysis of CML and RAGE protein expression with cardiac fibrosis (**A**, **C**) and inflammatory infiltrate (**B**, **D**). Pearson correlation of CML, RAGE protein expression and fluorescent AGEs with malondialdehyde content (**E**-**G**) and enzymatic activity of catalase (**H**-**J**). Representative Pearson correlation matrix of CML, RAGE protein expression and fluorescent AGEs with quantification of Th1, Th2, and Th17 cytokine and chemokine levels (**K**)

Pearson’s correlation analyses were performed to evaluate the role of AGEs in the development of cardiac pathological changes in T2D (Fig. 3A-J). These analyses revealed a strong positive correlation between CML and cardiac RAGE levels and cardiac fibrosis (CML: *r* = 0.8433, *p* = 0.001; RAGE: *r* = 0.7967, *p* = 0.001) (Fig. 3A and C) and a moderate positive correlation between CML and cardiac RAGE levels and cardiac inflammatory infiltrate (CML: *r* = 0.6387, *p* = 0.019; RAGE: *r* = 0.6502, *p* = 0.009) (Fig. 3B and D). For oxidative stress parameters, there was a strong positive correlation between cardiac RAGE and malondialdehyde levels (*r* = 0.7592, *p* = 0.001) (Fig. 3F), and a moderate positive correlation between fluorescent AGEs (*r* = 0.5609, *p* = 0.024) (Fig. 3G) and CML (*r* = 0.6884, *p* = 0.009)(Fig. 3E) with malondialdehyde levels. There was also a moderate positive correlation between cardiac CML (*r* = 0.6791, *p* = 0.011) (Fig. 3H), RAGE (*r* = 0.6169, *p* = 0.014)(Fig. 3I) and fluorescent AGEs (*r* = 0.5254, *p* = 0.037) (Fig. 3J) with catalase activity. In addition, the correlation between the assessed cytokines and AGE pathway parameters was plotted in matrix form (Fig. 3K). There was a strong positive correlation between cardiac CML levels and IL-10 (*r* = 0.76, *p* = 0.002) and IL-6 (*r* = 0.88, *p* = 0.005), and a moderate correlation with IL-17 A (*r* = 0.70, *p* = 0.008), TNF- α (*r* = 0.61, *p* = 0.026), IFN-γ (*r* = 0.64, *p* = 0.017), IL-4 (*r* = 0.61, *p* = 0.025) and IL-2 (*r* = 0.57, *p* = 0.044). In contrast, RAGE showed a moderate positive correlation with IL-6 (*r* = 0.60, *p* = 0.018) and IL-4 (*r* = 0.56, *p* = 0.029) (Fig. 3K).

## Discussion

T2D is a complex disease, and one of its main complications is an increased risk of CVDs, which are the leading cause of morbidity and mortality in this population [54]. Although the systemic benefits of exercise in T2D are well known, gaps remain regarding the mechanisms involved in modulating specific pathophysiological processes in cardiac tissue. In our study, we demonstrated that T2D mice exhibited activation of the AGE signaling pathway in cardiac tissue, with increased deposition of fluorescent AGEs, elevated protein expression of CML and RAGE, and higher gene expression of CD36 and galectin-3. Importantly, aerobic exercise reduced the expression of RAGE and CML as well as the levels of CD36 and galectin-3, indicating a direct effect of exercise on this signaling axis. The effects of aerobic exercise in mice with T2D were associated with decreased activation of inflammatory cascades and fibrosis in the heart, thus reducing the cardiac damage caused by diabetes.

Inflammation is central to the pathogenesis of cardiovascular complications in T2D, and we investigated whether the AGE signaling pathway could act as a key mediator of inflammation and tissue remodeling [55]. In our study, T2D mice showed markedly increased inflammatory infiltrates and fibrosis in cardiac tissue, accompanied by elevated levels of pro-inflammatory cytokines such as IL-6, TNF-α, NF-κB, and IL-10, as well as a reduction in cardioprotective markers. Aerobic exercise training attenuated these changes by reducing both inflammatory infiltration and fibrosis, and lowering cytokine levels together with the attenuation of several factors involved in the AGE signaling axis. This is consistent with previous studies reporting that, in the context of diabetes, AGEs, including fluorescent AGEs and CML, bind to RAGE and trigger the activation of intracellular signaling pathways that maintain a pro-inflammatory and pro-fibrogenic environment in the heart [27, 56, 57].

The AGE pathway, and its interactions exacerbate tissue stiffness, amplify inflammatory responses, and accelerate cardiac remodeling, which are hallmarks of cardiovascular complications in T2D. The increased RAGE expression observed in the hearts of diabetic mice which was counteracted by aerobic exercise underscores its contribution to a persistent inflammatory milieu in the cardiac microenvironment [58]. Similarly, CD36, another AGE-related receptor, promotes oxidative stress and fibrosis by facilitating the uptake of oxidized molecules [59, 60], and was also downregulated in exercised T2D mice. Furthermore, supporting the role of the AGE signaling pathway in the inflammatory response and tissue remodeling in T2D hearts, we observed a strong correlation between CML and RAGE levels and fibrosis, a moderate correlation with inflammatory infiltrates, and a strong correlation between RAGE and CML levels and pro-inflammatory cytokines. No significant modulation of GLO1 was observed, suggesting that the benefits of exercise in our model are mainly explained by changes in receptors and downstream signaling rather than AGE detoxification [61, 62].

In our study, exercise reduced the expression of galectin-3, an AGE-related receptor associated with fibrosis and inflammation, suggesting a new mechanism by which exercise could help mitigate cardiac pathological processes [62]. By reducing galectin-3, exercise likely impairs fibroblast activation and collagen deposition [63], which is consistent with the reduced cardiac fibrosis quantified by histopathology analyses. The attenuation of the inflammatory cascade and fibrosis triggered by exercise may contribute to the improvement of cardiac function [49, 64, 65]. Our functional cardiac and histological data together indicate that the receptor/cytokine/fibrosis triad was favorably influenced by training, supporting a causal chain from AGE pathway modulation to reduced inflammation and structural preservation. These results underscore the dual benefit of exercise: it not only lowers classic systemic inflammatory cascades but also directly affects the AGE signaling pathway and reduces its contribution to chronic inflammation and tissue remodeling in the heart.

Oxidative stress also plays a key role in the progression of cardiovascular complications in T2D [66, 67]. In our study, oxidative stress markers showed increased lipid peroxidation and a tendency toward higher catalase activity in diabetic hearts, indicating an imbalance between ROS production and antioxidant defense. However, despite this redox disturbance, aerobic training in our protocol did not produce significant changes in classical antioxidant enzymes, suggesting that the training effect was on the receptor/inflammation/fibrotic axis rather than direct upregulation of antioxidant enzymes. Persistent hyperglycemia and maintenance of the HFHC diet during the intervention may have limited redox adaptations, while the exercise dose (intensity/volume/duration) may have been below the threshold required to remodel cardiac-specific ROS sources [68–70]. The observed positive correlation between AGEs and markers of oxidative stress (MDA levels and CAT activity) suggests that the accumulation of AGEs may further exacerbate redox imbalance and potentially overwhelm cardiac tissue antioxidant defenses [71–75].

## Conclusions and future directions

This study shows that aerobic exercise provides cardioprotective effects in T2D by modulating the AGE signaling pathway, specifically through the downregulation of RAGE, CML, and galectin-3. These findings reveal a novel mechanism by which exercise reduces cardiac fibrosis and inflammation, highlighting the AGE axis as a key driver of diabetic cardiomyopathy and a promising therapeutic target. The benefits of exercise are mediated mainly by anti-inflammatory and anti-fibrotic effects rather than by reducing oxidative stress, positioning aerobic training as a strategic non-pharmacological intervention to limit cardiac complications in T2D.

Future directions include investigating sex-specific responses, optimizing exercise protocols, and testing combined strategies with pharmacological agents targeting the AGE pathway. In particular, combining exercise with antioxidants or AGE receptor inhibitors may synergistically reduce the harmful effects of this pathway and enhance treatment efficacy for diabetes-related cardiovascular complications.

### Limitations

Evidence from human and preclinical studies indicates pronounced sex differences in diabetic cardiomyopathy, with women exhibiting higher cardiovascular risk and reduced exercise capacity, highlighting the limitation of relying solely on male models [73, 74, 76]. Future studies should include both sexes to determine whether aerobic exercise differentially modulates cardiac remodeling and AGE signaling. Our analysis of oxidative stress, limited to MDA levels and CAT activity, may not fully capture redox complexity, and the absence of significant differences could reflect limited assay sensitivity. Additionally, the lack of direct cardiac function assessments (such as, echocardiography or hemodynamics) limits the ability to correlate molecular changes with functional outcomes.

Despite these limitations, our findings suggest that structured aerobic exercise programs targeting the AGE axis may serve as effective, nonpharmacological strategies to prevent or mitigate cardiac complications in T2D, reinforcing the potential of exercise as a personalized therapeutic tool in diabetes-related cardiovascular disease.

## Supplementary Information

Supplementary Material 1: Figure 1: Mechanistic representation of the AGE-RAGE signaling pathway in the context of type 2 diabetes (T2D). Hyperglycemia leads to increased glucose flux through metabolic pathways such as polyol, hexosamine and glycolysis, resulting in oxidative stress, mitochondrial dysfunction and activation of inflammatory signaling. The accumulation of advanced glycation end products (AGEs) and their interaction with the receptor for AGEs (RAGE) trigger downstream signaling pathways involving oxidative phosphorylation overload, ER stress, apoptosis and fibrosis. These mechanisms culminate in structural and functional changes in the heart, including myocardial inflammation and fibrosis and vascular myocardial dysfunction.

Supplementary Material 2: Figure 2: Cytokine profiling of the heart using the CBA Mouse Inflammation Th1/Th2/Th17 assay. The Cytokine Kit utilizes bead array technology to simultaneously detect multiple cytokine proteins in cardiac tissue samples. Seven bead populations with different fluorescence intensities were coated with specific capture antibodies for the proteins IL-2, IL-4, IL-6, IFN-γ, TNF, IL-17 A, and IL-10. First, a cytometer setup beads are used to adjust the device voltages and compensation settings, as shown in: (A) Setting gate “P1” containing a single bead population. (B) Different bead populations coated with specific antibodies for each cytokine, were incubated with samples or standards and a PE-conjugated detection antibody to form cytokine-bead sandwich complexes. (C) Bead populations were identified by their unique fluorescence intensities, and cytokine levels were calculated from the PE signal relative to the (D) standard curves. The representative dots plot of the experimental groups: (E) CTL; (F) T2D and (G) T2D EX.

## Data Availability

The datasets used and/or analyzed in the current study are available from the corresponding author upon reasonable request.

## Abbreviations

T2D: Type 2 diabetes mellitus
T2D EX: Type 2 diabetes mellitus exercise
AGE: Advanced glycation end products
RAGE: Receptor advanced glycation end products
ALE: Advanced lipid peroxidation and products
CTL: Control; HFHC: high-fat high-carbohydrate
IL-6: Interleukin 6
IL-2: Interleukin 2
IFN: Interferon
IL-4: Interleukin 4
IL-10: Interleukin 10
IL17A: Interleukin 17
HDL: High density lipoprotein
LDL: Low density lipoprotein
CPI: Cardioprotective index
HE: Hematoxylin and eosin
BSA: Bovine sérum albumin
CML: Carboxynethyllysine
DAB: Diaminobenzidine
BCA: Cytometric bead array
RT-PCR: Real time reverse transcriptase reaction followed by polymerase chain reaction
TBARS: Thiobarbituric acid reactive substance
MDA: Malondialdehyde
PBS: Phosphate buffered saline
BHT: Butylated hydroxytoluene
SOD: Superoxide dismutase
CAT: Catalase
ROS: Reactive oxygen species
TNF-α: Tumor necrosis factor-alpha
NF-kB: Nuclear factor kappa-B
GLO1: Glyoxalase 1
NO: Nitric oxide
TGF-β: Transforming growth factor-beta
MAPK: Mitogen-activated protein kinase
HbA1c: Glycated hemoglobin
DIO: Diet-induced obesity
NaOH: Sodium hydroxide
AU: Arbitrary units
H2O2: Hydrogen peroxide
PE: Fluorochrome phycoerythrin
DIAPH1: Diaphanous-related formin 1
GAPDH: Glyceraldehyde-3-phosphate dehydrogenase
LPO: Lipid peroxidation; PBS: phosphate buffered saline
KPE: Potassium phosphate buffer
UV: Ultravioleta light
U/mg protein: Units per milligram protein
SEM: Standart error mean
NOX2: NADPH oxidase isoform 2
NOX4: NADPH oxidase isoform 4
Nrf2: Nuclear factor erythroid 2-related factor 2
ARE: Antioxidant response element
GSH: Reduced glutathione
NADPH: Nicotinamide adenine dinucleotide phosphate

## References

[1] Zimmet P, Alberti KG, Magliano DJ, Bennett PH. Diabetes mellitus statistics on prevalence and mortality: facts and fallacies. Nat Rev Endocrinol. 2016;12(10):616–22. 27388988 10.1038/nrendo.2016.105

[2] Javeed N, Matveyenko AV. Circadian etiology of type 2 diabetes mellitus. Physiology. 2018;33(2):138–50. 29412061 10.1152/physiol.00003.2018 PMC5899235

[3] Stratton IM. Association of glycaemia with macrovascular and microvascular complications of type 2 diabetes (UKPDS 35): prospective observational study. BMJ. 2000;321(7258):405–12. 10938048 10.1136/bmj.321.7258.405 PMC27454

[4] Kenny HC, Abel ED. Heart failure in type 2 diabetes mellitus. Circ Res. 2019;124(1):121–41. 30605420 10.1161/CIRCRESAHA.118.311371 PMC6447311

[5] Allison MA, Masoudi FA, Bao H, Spatz ES, Fonarow GC. Diabetes Mellitus and Outcomes of Cardiac Resynchronization With Implantable Cardioverter-Defibrillator Therapy in Older Patients With Heart Failure. Circulation-arrhythmia Electrophysiol. 2016;9(8):e004132. 10.1161/CIRCEP.116.004132 27489243

[6] Baynes JW. Chemical modification of proteins by lipids in diabetes. Clin Chem Lab Med. 2003;41(9):1159–65. 14598865 10.1515/CCLM.2003.179

[7] Yamagishi S, Maeda S, Matsui T, Ueda S, Fukami K, Okuda S. Role of advanced glycation end products (AGEs) and oxidative stress in vascular complications in diabetes. Biochimica et biophysica acta (BBA) -. Gen Subj. 2012;1820(5):663–71. 10.1016/j.bbagen.2011.03.014 21440603

[8] SchächingerV, Britten MB, Zeiher AM. Prognostic impact of coronary vasodilator dysfunction on adverse long-term outcome of coronary heart disease. Circulation. 2000;101(16):1899–906. 10779454 10.1161/01.cir.101.16.1899

[9] Suwaidi JA, Hamasaki S, Higano ST, Nishimura RA, Holmes DR, Lerman A. Long-term follow-up of patients with mild coronary artery disease and endothelial dysfunction. Circulation. 2000;101(9):948–54. 10704159 10.1161/01.cir.101.9.948

[10] Zhao LM, Zhang W, Wang LP, Li GR, Deng XL. Advanced glycation end products promote proliferation of cardiac fibroblasts by upregulation of KCa3.1 channels. Pflugers Arch Eur J Physiol. 2012;464(6):613–21. 23053478 10.1007/s00424-012-1165-0

[11] Tsoporis JN, Shehla Izhar, Proteau G, Slaughter G, Parker TG. S100B-RAGE dependent VEGF secretion by cardiac myocytes induces myofibroblast proliferation. J Mol Cell Cardiol. 2011;52(2):464–73. 21889514 10.1016/j.yjmcc.2011.08.015

[12] Twarda-Clapa A, Olczak A, Białkowska AM, Koziołkiewicz M. Advanced glycation end-products (AGEs): formation, chemistry, classification, receptors, and diseases related to ages. Cells. 2022;11(8):1312. 35455991 10.3390/cells11081312 PMC9029922

[13] Hua W, Peng L, Chen X, Jiang X, Hu J, Jiang XH, et al. CD36-mediated podocyte lipotoxicity promotes foot process effacement. Open Med. 2024;19(1):20240918. 10.1515/med-2024-0918 PMC10996993 38584832

[14] Zhang X, Fan J, Li H, Chen C, Wang Y. CD36 signaling in diabetic cardiomyopathy. Aging Dis. 2021;12(3):826. 34094645 10.14336/AD.2020.1217 PMC8139204

[15] Jiménez-González S, Delgado-Valero B, Islas F, Romero-Miranda A, Luaces M, Ramchandani B, et al. The detrimental role of galectin-3 and endoplasmic reticulum stress in the cardiac consequences of myocardial ischemia in the context of obesity. FASEB J. 2024;38(14):e23818. 38989572 10.1096/fj.202400747R

[16] Seropian IM, Cassaglia P, Verónica Miksztowicz, González GE. Unraveling the role of galectin-3 in cardiac pathology and physiology. Front Physiol. 2023;18(14): 1304735 10.3389/fphys.2023.1304735 PMC10759241 38170009

[17] Aragonès G, Rowan S, Francisco SG, Whitcomb EA, Yang W, Perini-Villanueva G, et al. The glyoxalase system in age-related diseases: nutritional intervention as anti-ageing strategy. Cells. 2021;10(8):1852. 34440621 10.3390/cells10081852 PMC8393707

[18] Cely I, Blencowe M, Shu L, Diamante G, Ahn IS, Zhang G, et al. Glo1 reduction in mice results in age- and sex-dependent metabolic dysfunction. bioRxiv: preprint Serv biology. 2025; 01(24): 634754.

[19] Cai H, Harrison DG. Endothelial dysfunction in cardiovascular diseases: the role of oxidant stress. Circ Res. 2000;87(10):840–4. 11073878 10.1161/01.res.87.10.840

[20] Goldin A, Beckman JA, Schmidt AM, Creager MA. Adv Glycation End Prod Circulation. 2006;114(6):597–605. 10.1161/CIRCULATIONAHA.106.621854 16894049

[21] Barton M. Prevention and endothelial therapy of coronary artery disease. Curr Opin Pharmacol. 2013;13(2):226–41. 23742924 10.1016/j.coph.2013.05.005

[22] Guzik TJ, West NEJ, Black E, McDonald D, Ratnatunga C, Pillai R, et al. Vascular Superoxide Production by NAD(P)H Oxidase: association with endothelial dysfunction and clinical risk factors. Circul Res. 2000;86(9): E85-90. 10.1161/01.res.86.9.e85 10807876

[23] Feng Z, Du Z, Shu X, Zhu L, Wu J, Gao Q, et al. Role of RAGE in obesity-induced adipose tissue inflammation and insulin resistance. Cell Death Discov. 2021;7:305. 34686659 10.1038/s41420-021-00711-w PMC8536716

[24] Wang J, Cai W, Yu J, Liu H, He S, Zhu L et al. Dietary Advanced Glycation End Products Shift the Gut Microbiota Composition and Induce Insulin Resistance in Mice. Diabetes, metabolic syndrome and obesity: targets and therapy. 2022 Autumn;15:427–37. 10.2147/DMSO.S346411 PMC8857970 35210793

[25] Adeshara KA, Bangar N, Diwan AG, Tupe RS. Plasma glycation adducts and various RAGE isoforms are intricately associated with oxidative stress and inflammatory markers in type 2 diabetes patients with vascular complications. Diabetes & Metabolic Syndrome: Clinical Research & Reviews. 2022;16(3):102441. 10.1016/j.dsx.2022.102441 35247657

[26] Jing Cao, Zhang G, Liu Z, Xu Q, Li C, Cheng G, et al. Peroxidasin promotes diabetic vascular endothelial dysfunction induced by advanced glycation end products via NOX2/HOCl/Akt/eNOS pathway. Redox Biol. 2021;45:102031. 34116361 10.1016/j.redox.2021.102031 PMC8192873

[27] LeWinter MM, Taatjes D, Ashikaga T, Palmer B, Bishop N, VanBuren P, et al. Abundance, localization, and functional correlates of the advanced glycation end-product carboxymethyl lysine in human myocardium. Physiol Rep. 2017;5(20):e13462. 29066596 10.14814/phy2.13462 PMC5661230

[28] Yoshida N, Okumura K, Aso Y. High serum pentosidine concentrations are associated with increased arterial stiffness and thickness in patients with type 2 diabetes. Metab Clin Exp. 2005;54(3):345–50. 15736112 10.1016/j.metabol.2004.09.014

[29] Papadaki M, Kampaengsri T, Barrick SK, Campbell SG, von Lewinski D, Rainer PP, et al. Myofilament glycation in diabetes reduces contractility by inhibiting tropomyosin movement, is rescued by cMyBPC domains. J Mol Cell Cardiol. 2022;162:1–9. 34487755 10.1016/j.yjmcc.2021.08.012 PMC8766917

[30] Peng H, Sarwar Z, Yang XP, Peterson EL, Xu J, Janic B, et al. Profibrotic role for interleukin-4 in cardiac remodeling and dysfunction. Hypertension. 2015;66(3):582–9. 26195478 10.1161/HYPERTENSIONAHA.115.05627 PMC4685692

[31] Egawa T, Ogawa T, Takumi Yokokawa, Kido K, Mami Fujibayashi, Goto K, et al. Glycative stress and skeletal muscle dysfunctions: as an inducer of Exercise-Resistance. Glycative Stress Res. 2022;9(4):199–205.

[32] Janssens JV, Ma B, Brimble MA, Van Eyk JE, Delbridge LMD, Mellor KM. Cardiac troponins may be irreversibly modified by glycation: novel potential mechanisms of cardiac performance modulation. Sci Rep. 2018;8(1):16084. 30382112 10.1038/s41598-018-33886-x PMC6208411

[33] Hu P, Zhou H, Lu M, Dou L, Bo G, Wu J, et al. Autophagy plays a protective role in advanced glycation end product-induced apoptosis in cardiomyocytes. Cell Physiol Biochem. 2015;37(2):697–706. 26356261 10.1159/000430388

[34] Huo S, Wang Q, Shi W, Peng L, Jiang Y, Zhu M, et al. ATF3/SPI1/SLC31A1 signaling promotes Cuproptosis induced by advanced glycosylation end products in diabetic myocardial injury. Int J Mol Sci. 2023;24(2):1667. 36675183 10.3390/ijms24021667 PMC9862315

[35] Deluyker D, Evens L, Haesen S, Driesen RB, Kuster D, Verboven M, et al. Glycolaldehyde-Derived High-Molecular-Weight advanced glycation End-Products induce cardiac dysfunction through structural and functional remodeling of cardiomyocytes. Cell Physiol Biochem. 2020;54(5):809–24. 10.33594/000000271 32857934

[36] Carroll S, Dudfield M. What is the relationship between exercise and metabolic abnormalities? Sports Med. 2004;34(6):371–418. 15157122 10.2165/00007256-200434060-00004

[37] Amanat S, Ghahri S, Dianatinasab A, Fararouei M, Dianatinasab M. Exercise and type 2 diabetes. Phys Exerc Hum Health. 2020;1228(1):91–105. 10.1007/978-981-15-1792-1_6 32342452

[38] Patel H, Alkhawam H, Madanieh R, Shah N, Kosmas CE, Vittorio TJ. Aerobic vs anaerobic exercise training effects on the cardiovascular system. World J Cardiol. 2017;9(2):134. 28289526 10.4330/wjc.v9.i2.134 PMC5329739

[39] Cristián C-M, Nicolás Garrido-Muñoz, Bastián Alvear-Constanzo, Sofía Sanzana-Laurié, Artigas-Arias M, Alegría-Molina A et al. Effects of high-intensity interval training on lean mass, strength, and power of the lower limbs in healthy old and young people. Frontiers in Physiology. 2023;14. 10.3389/fphys.2023.1223069 PMC10565117 37829114

[40] Chang GR, Hou PH, Chen WK, Lin CT, Tsai HP, Mao FC. Exercise affects blood glucose levels and tissue chromium distribution in High-Fat Diet-Fed C57BL6 mice. Molecules. 2020;25(7):1658. 32260278 10.3390/molecules25071658 PMC7180458

[41] Zhang H, Simpson LK, Carbone NP, Hirshman MF, Nigro P, Vamvini M, et al. Moderate-intensity endurance training improves late phase β-cell function in adults with type 2 diabetes. iScience. 2023;26(7):107226. 37485354 10.1016/j.isci.2023.107226 PMC10362261

[42] Muscella A, Stefàno E, Marsigliante S. The effects of exercise training on lipid metabolism and coronary heart disease. Am J Physiol Heart Circ Physiol. 2020;319(1):H76–88. 32442027 10.1152/ajpheart.00708.2019

[43] Park W, Jung WS, Hong K, Kim YY, Kim SW, Park HY. Effects of moderate combined Resistance- and Aerobic-Exercise for 12 weeks on body Composition, cardiometabolic risk Factors, blood Pressure, arterial Stiffness, and physical Functions, among obese older men: A pilot study. Int J Environ Res Public Health. 2020;17(19):7233. 33022918 10.3390/ijerph17197233 PMC7579509

[44] Ahmadi A, Moheb-Mohammadi F, Navabi ZS, Dehghani M, Heydari H, Sajjadi F, et al. The effects of aerobic training, resistance training, combined training, and healthy eating recommendations on lipid profile and body mass index in overweight and obese children and adolescents: A randomized clinical trial. ARYA Atherosclerosis. 2020;16(5):226–34. 33889189 10.22122/arya.v16i5.1990 PMC8034757

[45] Marandi SM, Abadi NGB, Esfarjani F, Mojtahedi H, Ghasemi G. Effects of intensity of aerobics on body composition and blood lipid profile in obese/overweight females. Int J Prev Med. 2013;4(Suppl 1):S118–25. 23717761 PMC3665017

[46] Lino Rodrigues K, Vieira Dias Da Silva V, Nunes Goulart da Silva Pereira E, Rangel Silvares R, Peres de Araujo B, Eduardo, Flores I et al. E,. Aerobic Exercise Training Improves Microvascular Function and Oxidative Stress Parameters in Diet-Induced Type 2 Diabetic Mice. Diabetes, Metabolic Syndrome and Obesity: Targets and Therapy. 2022;15:2991–3005. 10.2147/DMSO.S365496 PMC9527816 36200064

[47] Ito D, Cao P, Kakihana T, Sato E, Suda C, Muroya Y et al. Chronic Running Exercise Alleviates Early Progression of Nephropathy with Upregulation of Nitric Oxide Synthases and Suppression of Glycation in Zucker Diabetic Rats. Sen U, editor. PLOS ONE. 2015;10(9):e0138037. 10.1371/journal.pone.0138037 PMC4574951 26379244

[48] Delbin MA, Paula A, Gisele K, Couto, Gustavo LV, Rossoni, Antunes E, et al. Interaction between advanced glycation end products formation and vascular responses in femoral and coronary arteries from exercised diabetic Rats. Europe PMC (PubMed. Central). 2012;7(12):e53318–8. 10.1371/journal.pone.0053318 PMC3532341 23285277

[49] Gu Q, Bing W, Zhang X, Ma Y, Liu J, Wang X. Contribution of receptor for advanced glycation end products to vasculature-protecting effects of exercise training in aged rats. Eur J Pharmacol. 2014;741:186–94. 25160740 10.1016/j.ejphar.2014.08.017

[50] Malin SK, Navaneethan SD, Fealy CE, Scelsi A, Huang H, Rocco M, et al. Exercise plus caloric restriction lowers soluble RAGE in adults with chronic kidney disease. Obesity science & practice. 2020;6(3):307–12. 32523720 10.1002/osp4.408 PMC7278900

[51] Wang Y, Zhou Y, Jiang L, Wang S, Zhu L, Zhang S, et al. Long-term voluntary exercise inhibited AGE/RAGE and microglial activation and reduced the loss of dendritic spines in the hippocampi of APP/PS1 Transgenic mice. Exp Neurol. 2023;363:114371–1. 36871860 10.1016/j.expneurol.2023.114371

[52] Oršolić N, Landeka Jurčević I, Đikić D, Rogić D, Odeh D, Balta V, et al. Effect of propolis on diet-induced hyperlipidemia and atherogenic indices in mice. Antioxidants. 2019;8(6):156. 31163593 10.3390/antiox8060156 PMC6617317

[53] Nakayama H, Mitsuhashi T, Satoru Kuwajima, Aoki S, Kuroda Y, Itoh T, et al. Immunochemical detection of advanced glycation end products in lens crystallins from Streptozocin-induced diabetic rat. Diabetes. 1993;42(2):345–50. 8425672 10.2337/diab.42.2.345

[54] Tomás CC, Oliveira E, Sousa D, Uba-Chupel M, Furtado G, Rocha C et al. Proceedings of the 3rd IPLeiria’s International Health Congress. BMC Health Services Research. 2016;16(S3). 10.1186/s12913-016-1423-5 PMC4943498 27409075

[55] Rohm TV, Meier DT, Olefsky JM, Donath MY. Inflammation in obesity, diabetes, and related disorders. Immunity. 2022;55(1):31–55. 35021057 10.1016/j.immuni.2021.12.013 PMC8773457

[56] Rojas A, Lindner C, Schneider I, Gonzalez I, Uribarri J. The RAGE axis: a relevant inflammatory hub in human diseases. Biomolecules. 2024;14(4):412. 38672429 10.3390/biom14040412 PMC11048448

[57] Zhang H, Dhalla NS. The role of Pro-Inflammatory cytokines in the pathogenesis of cardiovascular disease. Int J Mol Sci. 2024;25(2):1082–2. 38256155 10.3390/ijms25021082 PMC10817020

[58] Lee TW, Kao YH, Lee TI, Chang CJ, Lien GS, Chen YJ. Calcitriol modulates receptor for advanced glycation end products (RAGE) in diabetic hearts. Int J Cardiol. 2014;173(2):236–41. 24630381 10.1016/j.ijcard.2014.02.041

[59] Okamura DM, Subramaniam Pennathur, Pasichnyk K, López-Guisa JM, Collins S, Febbraio M, et al. CD36 regulates oxidative stress and inflammation in hypercholesterolemic CKD. J Am Soc Nephrol. 2009;20(3):495–505. 19211715 10.1681/ASN.2008010009 PMC2653683

[60] Yang P, Xiao Y, Luo X, Zhao Y, Zhao L, Wang Y, et al. Inflammatory stress promotes the development of obesity-related chronic kidney disease via CD36 in mice. J Lipid Res. 2017;58(7):1417–27. 28536108 10.1194/jlr.M076216 PMC5496038

[61] Blackburn NJR, Vulesevic B, McNeill B, Cimenci CE, Ahmadi A, Gonzalez-Gomez M, et al. Methylglyoxal-derived advanced glycation end products contribute to negative cardiac remodeling and dysfunction post-myocardial infarction. Basic Res Cardiol. 2017. 10.1007/s00395-017-0646-x. 28864889 10.1007/s00395-017-0646-x

[62] Saeed M, Kausar MA, Singh R, Siddiqui AJ, Akhter A. The role of glyoxalase in glycation and carbonyl stress induced metabolic disorders. Curr Protein Pept Sci. 2020;21(9):846–59. 32368974 10.2174/1389203721666200505101734

[63] Henderson NC, Mackinnon AC, Farnworth SL, Poirier F, Russo FP, Iredale JP et al. Galectin-3 regulates myofibroblast activation and hepatic fibrosis. Proceedings of the National Academy of Sciences. 2006;103(13):5060–5. 10.1073/pnas.0511167103 PMC1458794 16549783

[64] Pedersen BK. Anti-inflammatory effects of exercise: role in diabetes and cardiovascular disease. Eur J Clin Invest. 2017;47(8):600–11. 28722106 10.1111/eci.12781

[65] Ludzki AC, Krueger EM, Baldwin TC, Schleh MW, Porsche CE, Ryan BJ et al. Acute aerobic exercise remodels the adipose tissue progenitor cell phenotype in obese adults. Front Physiol. 2020;11:903. 10.3389/fphys.2020.00903 PMC7399179 32848853

[66] De Rosa S, Arcidiacono B, Chiefari E, Brunetti A, Indolfi C, Foti DP. Type 2 diabetes mellitus and cardiovascular disease: genetic and epigenetic links. Front Endocrinol. 2018;9(2):2. 10.3389/fendo.2018.00002 PMC5776102 29387042

[67] Vassalle C, Gaggini M. Type 2 diabetes and oxidative stress and inflammation: pathophysiological mechanisms and possible therapeutic options. Antioxidants. 2022;11(5):953. 35624817 10.3390/antiox11050953 PMC9137541

[68] Atalay M, Laaksonen DE. Diabetes, oxidative stress and physical exercise. J Sports Sci Med. 2002;1(1):1–14. 24672266 PMC3957575

[69] Ni C, Ji Y, Hu K, Xing K, Xu Y, Gao Y. Effect of exercise and antioxidant supplementation on cellular lipid peroxidation in elderly individuals: systematic review and network meta-analysis. Front Physiol. 2023;14. 10.3389/fphys.2023.1113270 PMC9971974 36866175

[70] Crane JD, Abadi A, Hettinga BP, Ogborn DI, MacNeil LG, Steinberg GR et al. Elevated Mitochondrial Oxidative Stress Impairs Metabolic Adaptations to Exercise in Skeletal Muscle. Moro C, editor. PLoS ONE. 2013;8(12):e81879. 10.1371/journal.pone.0081879 PMC3855701 24324727

[71] Qi W, Niu J, Qin Q, Qiao Z, Gu Y. Glycated albumin triggers fibrosis and apoptosis via an NADPH oxidase/Nox4-MAPK pathway-dependent mechanism in renal proximal tubular cells. Molecular and cellular endocrinology. 2015 Autumn;405:74–83. 10.1016/j.mce.2015.02.007 25681565

[72] Lin MT, Yin R, Yang L, Zhao D. Role of advanced glycation end products on vascular smooth muscle cells under diabetic atherosclerosis. Front Endocrinol. 2022; 31(13) :983723. 10.3389/fendo.2022.983723 PMC9470882 36120471

[73] Chandramouli C, Reichelt ME, Curl CL, Varma U, Bienvenu LA, Koutsifeli P, et al. Diastolic dysfunction is more apparent in STZ-induced diabetic female mice, despite less pronounced hyperglycemia. Sci Rep. 2018;8(1):2346. 10.1038/s41598-018-20703-8 PMC5799292 29402990

[74] REGENSTEINER JG, BAUER TA, HUEBSCHMANN AG, WEINBERGER HERLACHEL, WOLFEL HD. Sex differences in the effects of type 2 diabetes on exercise performance. Med Sci Sports Exerc. 2015;47(1):58–65. 24811327 10.1249/MSS.0000000000000371 PMC4296732

[75] Tang X, Jiang S, Zhang J, Zhou S, Zheng Y. Sex Differences in Progression of Diabetic Cardiomyopathy in OVE26 Type 1 Diabetic Mice. Oxidative medicine and cellular longevity. 2020 Summer; 2020:6961348. 10.1155/2020/6961348 PMC7244980 32509150

[76] Tang X, Jiang S, Zhang J, Zhou S, Zheng Y. Sex differences in progression of diabetic cardiomyopathy in OVE26 type 1 diabetic mice. Oxidative Med Cell Longev. 2020;2020(Summer):6961348. 10.1155/2020/6961348 PMC7244980 32509150

