# Supplementary figures and images for: Aerobic exercise training attenuates cardiac inflammation and fibrosis in mice with type 2 diabetes and inhibits the advanced glycation end products pathway
Source: Diabetol Metab Syndr. 2026 Jan 7;18:46. doi: 10.1186/s13098-025-02076-x (PMC12870502; doi:10.1186/s13098-025-02076-x)

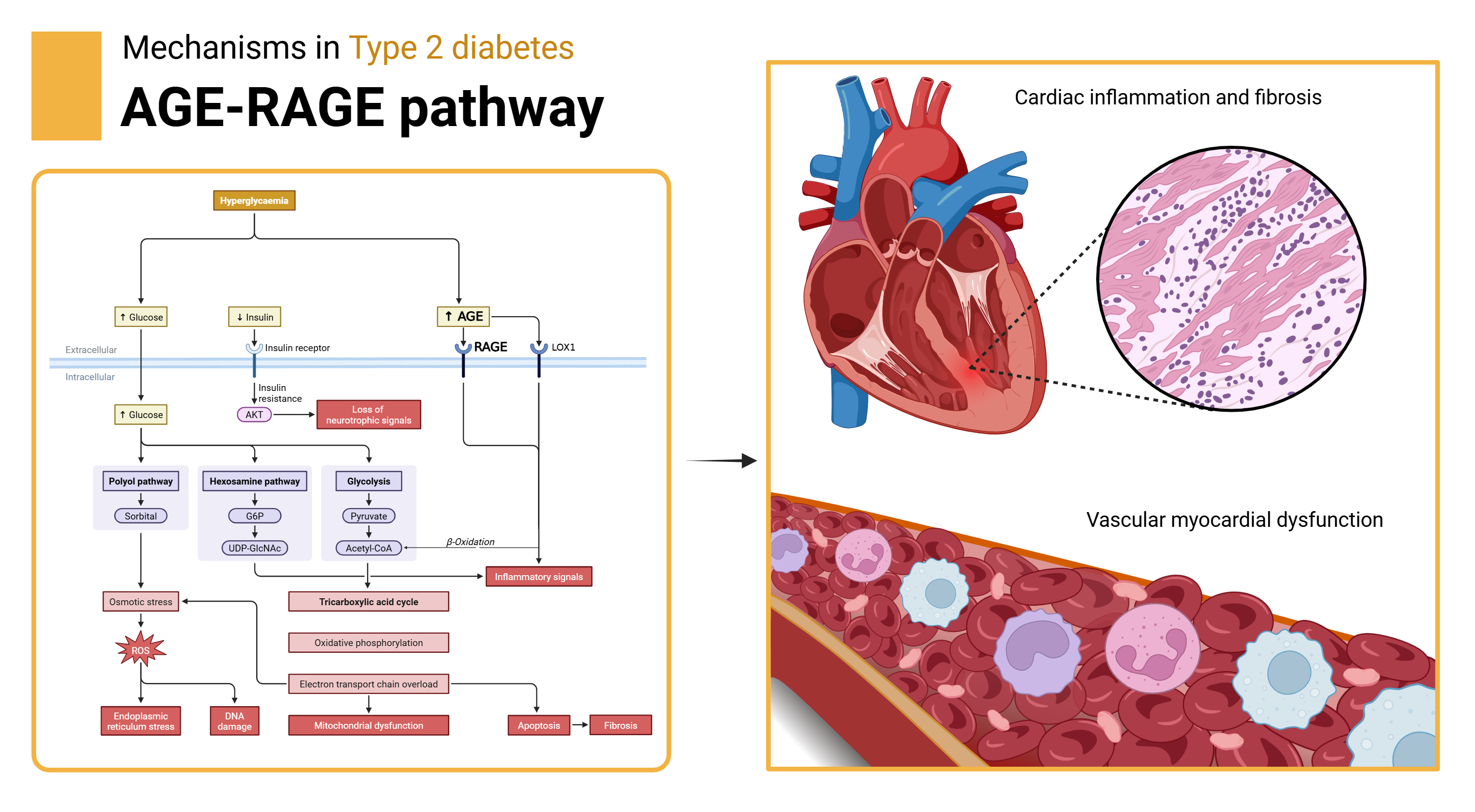

Supplement: Supplementary file 1 — Supplementary Material 1: Figure 1: Mechanistic representation of the AGE-RAGE signaling pathway in the context of type 2 diabetes (T2D). Hyperglycemia leads to increased glucose flux through metabolic pathways such as polyol, hexosamine and glycolysis, resulting in oxidative stress, mitochondrial dysfunction and activation of inflammatory signaling. The accumulation of advanced glycation end products (AGEs) and their interaction with the receptor for AGEs (RAGE) trigger downstream signaling pathways involving oxidative phosphorylation overload, ER stress, apoptosis and fibrosis. These mechanisms culminate in structural and functional changes in the heart, including myocardial inflammation and fibrosis and vascular myocardial dysfunction. [file 13098_2025_2076_MOESM1_ESM.png]

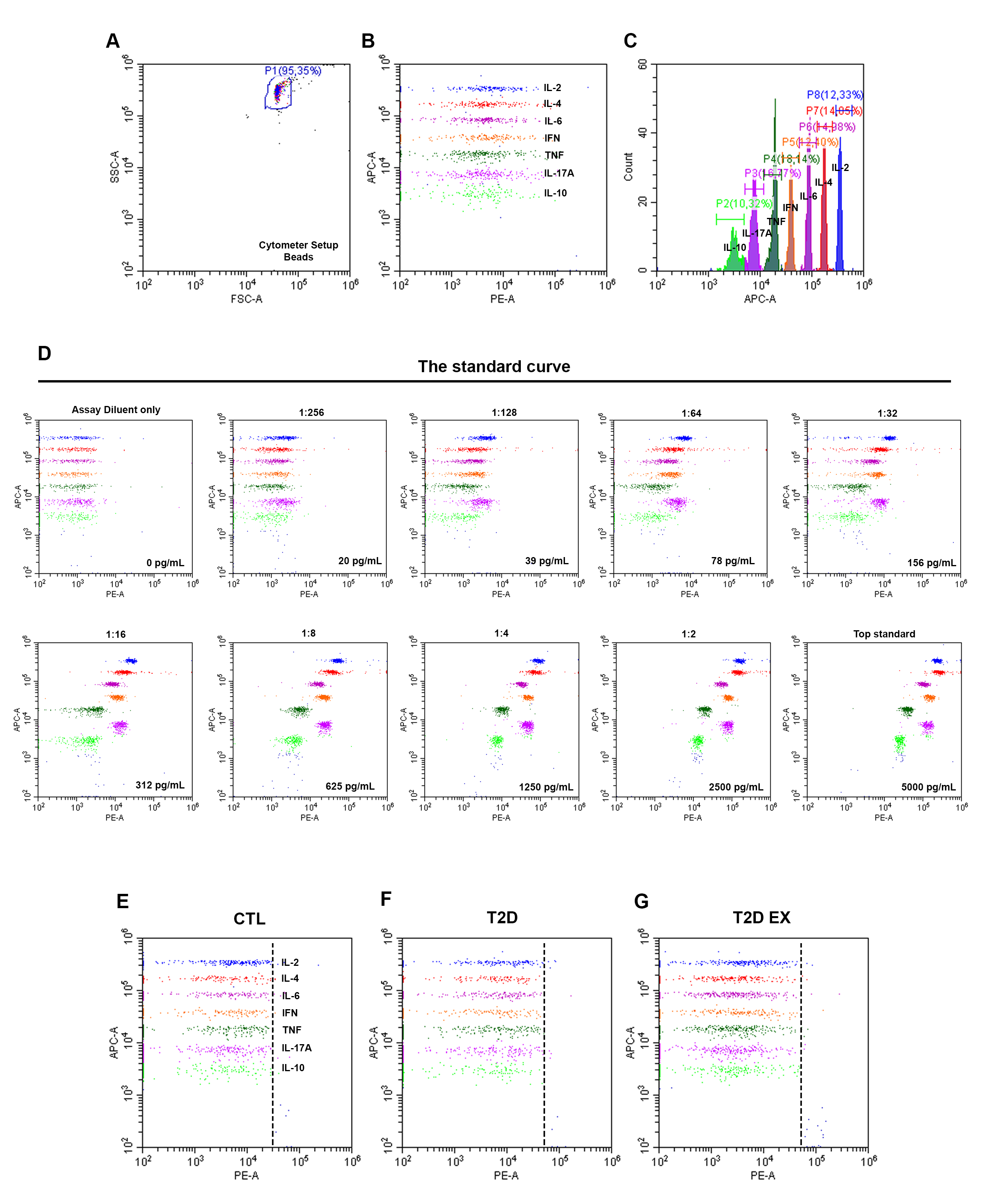

Supplement: Supplementary file 2 — Supplementary Material 2: Figure 2: Cytokine profiling of the heart using the CBA Mouse Inflammation Th1/Th2/Th17 assay. The Cytokine Kit utilizes bead array technology to simultaneously detect multiple cytokine proteins in cardiac tissue samples. Seven bead populations with different fluorescence intensities were coated with specific capture antibodies for the proteins IL-2, IL-4, IL-6, IFN-γ, TNF, IL-17 A, and IL-10. First, a cytometer setup beads are used to adjust the device voltages and compensation settings, as shown in: (A) Setting gate “P1” containing a single bead population. (B) Different bead populations coated with specific antibodies for each cytokine, were incubated with samples or standards and a PE-conjugated detection antibody to form cytokine-bead sandwich complexes. (C) Bead populations were identified by their unique fluorescence intensities, and cytokine levels were calculated from the PE signal relative to the (D) standard curves. The representative dots plot of the experimental groups: (E) CTL; (F) T2D and (G) T2D EX. [file 13098_2025_2076_MOESM2_ESM.tif]
